# Genetic polymorphisms of *PKLR* gene and their associations with milk production traits in Chinese Holstein cows

**DOI:** 10.3389/fgene.2022.1002706

**Published:** 2022-09-02

**Authors:** Aixia Du, Fengru Zhao, Yanan Liu, Lingna Xu, Kewei Chen, Dongxiao Sun, Bo Han

**Affiliations:** ^1^ National Engineering Laboratory of Animal Breeding, Key Laboratory of Animal Genetics, Department of Animal Genetics and Breeding, Breeding and Reproduction of Ministry of Agriculture and Rural Affairs, College of Animal Science and Technology, China Agricultural University, Beijing, China; ^2^ Beijing Dairy Cattle Center, Beijing, China; ^3^ Yantai Institute, China Agricultural University, Yantai, China

**Keywords:** PKLR, milk production traits, association analysis, GS, SNP chips

## Abstract

Our previous work had confirmed that pyruvate kinase L/R (*PKLR*) gene was expressed differently in different lactation periods of dairy cattle, and participated in lipid metabolism through insulin, PI3K-Akt, MAPK, AMPK, mTOR, and PPAR signaling pathways, suggesting that *PKLR* is a candidate gene to affect milk production traits in dairy cattle. Here, we verified whether this gene has significant genetic association with milk yield and composition traits in a Chinese Holstein cow population. In total, we identified 21 single nucleotide polymorphisms (SNPs) by resequencing the entire coding region and partial flanking region of *PKLR* gene, in which, two SNPs were located in 5′ promoter region, two in 5′ untranslated region (UTR), three in introns, five in exons, six in 3′ UTR and three in 3′ flanking region. The single marker association analysis displayed that all SNPs were significantly associated with milk yield, fat and protein yields or protein percentage (*p* ≤ 0.0497). The haplotype block containing all the SNPs, predicted by Haploview, had a significant association with fat yield and protein percentage (*p* ≤ 0.0145). Further, four SNPs in 5′ regulatory region and eight SNPs in UTR and exon regions were predicted to change the transcription factor binding sites (TFBSs) and mRNA secondary structure, respectively, thus affecting the expression of *PKLR*, leading to changes in milk production phenotypes, suggesting that these SNPs might be the potential functional mutations for milk production traits in dairy cattle. In conclusion, we demonstrated that *PKLR* had significant genetic effects on milk production traits, and the SNPs with significant genetic effects could be used as candidate genetic markers for genomic selection (GS) in dairy cattle.

## Introduction

Milk is rich in nutrition and is an important food for the human body to obtain many essential nutrients. Fat and protein in milk have the characteristics of easy digestion and absorption, especially for children and the elderly, so the content and proportion of fat and protein in milk is of great significance. Studies have shown that drinking milk can reduce the incidence of dental caries (Rumbold et al., 2021), cardiovascular disease (Soedamah-Muthu and de Goede 2018), metabolic syndrome (Crichton et al., 2011) and obesity (Abargouei et al., 2012). Dairy cattle breeding is essential for the development of the dairy industry and human health. In dairy cattle breeding, one of the most important thing is to study the milk production traits, milk yield, fat yield, and percentage, and protein yield and percentage, which are quantitative traits and controlled by multiple minor polygenes, a few main efficient genes and greatly affected by the environment (Schrooten et al., 2000). However, the process of traditional breeding is very slow and unable to meet the growing consumer demand.

Meuwissen et al. (2001) first proposed genomic selection (GS) in 2001, which can better reflect the problem of minorgenes for quantitative traits (Wiggans et al., 2011). Especially for animals such as dairy cattle with long generation interval, GS can effectively shorten their generation interval and accelerate genetic progress (Stock and Reents 2013). Since 2009, GS has been formally applied to dairy cattle breeding, which has brought revolutionary changes to dairy cattle breeding (Wiggans et al., 2017). SNP (single nucleotide polymorphism) chips designed with SNP probes based on large-scale SNP genotype data to detect genomic polymorphism (Heffner et al., 2009) were used in GS to select target traits. In recent years, with the development of SNP chip technology, GS has been widely used in dairy cattle breeding (Jiang et al., 2013; Jiang et al., 2016). Through GS, a single marker whose effect is small can be captured (Goddard and Hayes 2007). Additionally, studies have shown that adding functional site information with large genetic effects on target traits can improve the accuracy of GS (Zhang et al., 2014; Brondum et al., 2015; Zhang et al., 2015; de Las Heras-Saldana et al., 2020). Therefore, in recent years, researchers have been using various methods such as quantitative trait locus (QTL) mapping, candidate gene analysis, genome-wide association study (GWAS) and high throughput omics strategy to explore functional genes and mutations related to milk production traits, so as to improve the accuracy of GS and accelerate the process of molecular breeding of dairy cattle (Gebreyesus et al., 2019; Lopdell et al., 2019; Liu et al., 2020; Korkuc et al., 2021). At present, in terms of milk producing traits of dairy cattle, many genes such as *CDKN1A*, *FADS2*, *PRLR*, *SLC2A12,* and *SLC5A1* had been verified to be associated with milk yield and composition traits of Holstein cows (Maryam et al., 2015; Han et al., 2017; Yan et al., 2018; Shi et al., 2019; Valsalan et al., 2021; Zwierzchowski et al., 2021; Fu et al., 2022).

Previously, we obtained liver transcriptome data of Chinese Holstein cows at different lactations, and found that pyruvate kinase L/R (*PKLR*) gene was differentially expressed during periods and participated in lipid metabolism through insulin, PI3K-Akt, MAPK, AMPK, mTOR, and PPAR signaling pathways, suggesting that *PKLR* gene may play an important role for milk fat trait of dairy cattle (Liang et al., 2017). *PKLR* is involved in glycogen and lipid metabolisms in liver tissues (Wang et al., 2000; Ahrens et al., 2013), and has a wide association with a spectrum of liver damage from steatosis and inflammation to fibrosis *via* its regulation on mitochondrial dysfunction and subsequent hepatic triglyceride accumulation (Chella Krishnan et al., 2021). In addition, *PKLR* (chr.3: 15344765-15354042) is located 0.02 Mb to the peak of QTL regions for milk fat percentage (QTL_ID: 104486) and protein percentage (QTL_ID:104816, 104938) (Nayeri et al., 2016). Therefore, we considered this gene to be a potential candidate gene for milk producing traits in dairy cows.

Herein, we identified SNPs of the *PKLR* gene in a Chinese Holstein population and analyzed their genetic associations with milk yield, fat yield, fat percentage, protein yield and protein percentage. Further, we predicted the potential biological effects of identified SNPs on transcription factor binding site (TFBS) and mRNA secondary structure. The purpose of this study is to provide valuable SNP loci information for dairy GS, and also to provide some reference information for the in-depth study of the mechanism of candidate genes related to milk production traits in dairy cattle.

## Materials and methods

### Animals and phenotypic data

In this study, we used a total of 925 Chinese Holstein cows from 44 sire families for association analyses, and these cows were distributed in 21 dairy farms belonging to the Beijing Shounong Animal Husbandry Development Co., Ltd. (Beijing, China), where the cows were healthy with the same feeding conditions and had accurate pedigree information and standard dairy herd improvement (DHI) records. We used the phenotypic data of 925 cows in the first lactation and 633 in the second lactation (292 cows merely completed the milking of first lactation) for the association analyses and mainly analyzed five milk production traits, including 305-days milk yield, fat yield, fat percentage, protein yield and protein percentage. The descriptive statistics of phenotypic values for dairy production traits of the first and second lactations were presented in Supplementary Table S1.

### DNA extraction

The Beijing Dairy Cattle Center (Beijing, China) provides frozen semen of the 44 bulls and blood samples of 925 cows that were stored at −20°C for genomic DNA extraction. We extracted frozen semen DNAs by salt-out procedure, and extracted DNAs of blood samples by a TIANamp Blood DNA Kit (Tiangen, Beijing, China). Then, we used NanoDrop 2000 Spectrophotometer (Thermo Scientific, Hudson, NH, United States) and the gel electrophoresis to determine the quantity and quality of the extracted DNAs, respectively.

### SNP identification and genotyping

According to the sequences of bovine *PKLR* gene (NC_037330) from GenBank (https://www.ncbi.nlm.nih.gov/genbank/), we used Primers3 (https://primer3.ut.ee/) to design the primers (Supplementary Table S2) in this gene’s coding region, parts of intron region and 2,000 bp of upstream and downstream regions. The primers were synthesized by Beijing Genomics Institute (BGI, Beijing, China). We mixed the semen DNAs equally, amplified them by PCR (Supplementary Table S3), and detected the PCR amplification products using 2% gel electrophoresis before Sanger sequencing by BGI. After sequencing, we identified the potential SNPs according to the reference sequences (ARS-UCD1.2) on NCBI-BLAST (https://blast.ncbi.nlm.nih.gov/Blast.cgi). Subsequently, we genotyped the identified SNPs in 925 cows using Genotyping by Target Sequencing (GBTS) technology by Boruidi Biotechnology Co., Ltd. (Hebei, China).

### Linkage disequilibrium estimation and association analyses

We used Haploview4.2 (Broad Institute of MIT and Harvard, Cambridge, MA, United States) to estimate the extent of linkage disequilibrium (LD) between the identified SNPs.

The MIXED process in SAS 9.4 (SAS Institute Inc., Cary, NC, United States) software was used to carry out association analyses between the genotypes/haplotype blocks and the five milk production traits, milk yield, fat yield, fat percentage, protein yield, and protein percentage, on the first and second lactations. The following animal model was used for the association analysis: y=μ+HYS+b×M+G+a+e ; where y is the phenotypic value of each trait for each cow; µ is the overall mean; HYS is the fixed effect of farm (1–21 for 21 farms, respectively), year (1–4 for the year 2012–2015, respectively), and season (1 for April–May; 2 for June–August; 3 for September–November; and 4 for December– March); M is the age of calving as a covariant, b is the regression coefficient of covariant M; G is the genotype or haplotype combination effect; a is the individual random additive genetic effect, distributed as $N\;\left(0,\;A\delta_{a}^{2}\right)$ , with the additive genetic variance $\delta_{a}^{2}$ ; and e is the random residual, distributed as $N\;\left(0,\;I\delta_{e}^{2}\right)$ , with identity matrix I and residual error variance $\delta_{e}^{2}$ .

Additionally, we calculated the additive effect (a), dominant effect (d), and substitution effect (α) by the following formulas: $a=\frac{AA-BB}{2}$ , $d=AB-\frac{AA+BB}{2}$ , α=a+d(q−p) , where AA, BB and AB are the least square means of the milk production traits in the corresponding genotypes, *p* is the frequency of allele A, and q is the frequency of allele B.

### Functional prediction of mutation sites

We predicted changes of TFBSs for the SNPs located in the 5′ region of *PKLR* gene by the MEME Suite (http://meme-suite.org/). We used RNAfold Web Server (http://rna.tbi.univie.ac.at/cgi-bin/RNAWebSuite/RNAfold.cgi) to predict changes in secondary structures of mRNA for SNPs in UTR and exon regions. The minimum free energy (MFE) of the optimal secondary structure reflects the stability of mRNA structure. The lower the MFE value, the more stable the mRNA structure is.

## Results

### SNPs identification

In this study, we totally found 21 SNPs in *PKLR* gene, all of which had been reported previously. Two SNPs, 3:g.15342877C>T and 3:g.15344349A>C, were located in 5′ promoter region, two (3:g.15345216C>T and 3:g.15345227T>C) in 5′ untranslated region (UTR), three (3:g.15349740A>G, 3:g.15350548C>T and 3:g.15350805T>C) in introns, five (3:g.15349768A>G, 3:g.15349978A>G, 3:g.15350655A>G, 3:g.15350898T>C and 3:g.15352855T>C) in exons, six (3:g.15353088A>C, 3:g.15353235T>C, 3:g.15353254T>C, 3:g.15353292C>G, 3:g.15353330A>G and 3:g.15353342C>T) in 3′ UTR, and three (3:g.15355389T>C, 3:g.15355514T>C and 3:g.15355833A>G) in 3′ flanking region. All the five SNPs in the exons were synonymous mutations (Table 1). The genotypic and allelic frequencies of all the identified SNPs were summarized in Table 1.

**TABLE 1:** Details of SNPs identified in *PKLR* gene.

SNP name	GenBank no.	Location	Genotype	Genotypic frequency	Allele	Allelic frequency
3:g.15342877C>T	rs134381383	5′ promoter region	CC	0.0724	C	0.2876
			CT	0.4303	T	0.7124
			TT	0.4973		
3:g.15344349A>C	rs135669860	5′ promoter region	AA	0.0724	A	0.287
			AC	0.4292	C	0.713
			CC	0.4984		
3:g.15345216C>T	rs134794841	5′ UTR	CC	0.0724	C	0.287
			CT	0.4292	T	0.713
			TT	0.4984		
3:g.15345227T>C	rs110280638	5′ UTR	CC	0.4995	C	0.7135
			CT	0.4281	T	0.2865
			TT	0.0724		
3:g.15349740A>G	rs109049992	intron	AA	0.0714	A	0.2865
			AG	0.4303	G	0.7135
			GG	0.4984		
3:g.15349768A>G	rs110522117	exon 7	AA	0.0714	A	0.2865
			AG	0.4303	G	0.7135
			GG	0.4984		
3:g.15349978A>G	rs109620290	exon 7	AA	0.0714	A	0.2859
			AG	0.4292	G	0.7141
			GG	0.4994		
3:g.15350548C>T	rs109009333	intron	CC	0.0714	C	0.2865
			CT	0.4303	T	0.7135
			TT	0.4984		
3:g.15350655A>G	rs135555311	exon 9	AA	0.0714	A	0.2865
			AG	0.4303	G	0.7135
			GG	0.4984		
3:g.15350805T>C	rs109578013	intron	CC	0.4984	C	0.7135
			CT	0.4303	T	0.2865
			TT	0.0714		
3:g.15350898T>C	rs208110429	exon 10	CC	0.0281	C	0.1827
			CT	0.3092	T	0.8173
			TT	0.6627		
3:g.15352855T>C	rs109938041	exon 12	CC	0.4984	C	0.7135
			CT	0.4303	T	0.2865
			TT	0.0714		
3:g.15353088A>C	rs135526735	3′ UTR	AA	0.0714	A	0.287
			AC	0.4313	C	0.713
			CC	0.4973		
3:g.15353235T>C	rs109536098	3′ UTR	CC	0.4951	C	0.7114
			CT	0.4324	T	0.2886
			TT	0.0724		
3:g.15353254T>C	rs110474872	3′ UTR	CC	0.4951	C	0.7114
			CT	0.4324	T	0.2886
			TT	0.0724		
3:g.15353292C>G	rs136694042	3′ UTR	CC	0.0757	C	0.2908
			CG	0.4303	G	0.7092
			GG	0.494		
3:g.15353330A>G	rs135489031	3′ UTR	AA	0.0854	A	0.2957
			AG	0.4205	G	0.7043
			GG	0.4941		
3:g.15353342C>T	rs133320650	3′ UTR	CC	0.0886	C	0.2978
			CT	0.4184	T	0.7022
			TT	0.493		
3:g.15355389T>C	rs133757664	3′ flanking region	CC	0.4935	C	0.6876
			CT	0.3883	T	0.3124
			TT	0.1182		
3:g.15355514T>C	rs132659643	3′ flanking region	CC	0.4951	C	0.6891
			CT	0.3879	T	0.3109
			TT	0.117		
3:g.15355833A>G	rs108993332	3′ flanking region	AA	0.1159	A	0.3099
			AG	0.3879	G	0.6901
			GG	0.4962		

Note: UTR: untranslated region.

### Associations between SNPs and five milk productions traits

We analyzed the associations between the 21 SNPs in *PKLR* and five milk production traits in dairy cattle. In the first lactation, there were four, nineteen, four and seventeen SNPs significantly associated with milk yield, fat yield, protein yield and protein percentage, respectively (*p* ≤ 0.0497; Table 2). Four SNPs, 3:g.15350898T>C, 3:g.15355389T>C, 3:g.15355514T>C and 3:g.15355833A>G, had extremely significant genetic effects on milk, fat and protein yields (*p* ≤ 0.0044), and 3:g.15355389T>C and 3:g.15355514T>C were also significantly associated with protein percentage (*p* ≤ 0.0374). As for the second lactation, there were sixteen, twenty and eighteen SNPs were significantly associated with milk yield, fat yield and protein percentage (*p* ≤ 0.0436), respectively. Additionally, thirteen SNPs were significantly associated with milk yield, fat yield and protein percentage (*p* ≤ 0.0063). During two lactation periods, six SNPs, 3:g.15353292C>G, 3:g.15353330A>G, 3:g.15353342C>T, 3:g.15355389T>C, 3:g.15355514T>C and 3:g.15355833A>G, had significant genetic effects on fat yield (*p* ≤ 0.0097). In addition, the results of allelic additive, dominant and substitution effects of the SNPs in *PKLR* gene were displayed in Supplementary Table S4.

**TABLE 2:** Associations of 21 SNPs in *PKLR* with milk production traits in two lactations of Chinese Holstein cows (LSM ±SE).

SNP name	Lactation	Genotype (No.)	Milk yield (kg)	Fat yield (kg)	Fat percentage (%)	Protein yield (kg)	Protein percentage (%)
3:g.15342877C>T	1	CC (67)	9,994.06 ± 192.05	325.52 ± 8.0346^AB^	3.2752 ± 0.07772	299.96 ± 5.857	3.0133 ± 0.02668^a^
		CT (398)	10014 ± 179.5	327.21 ± 7.597^A^	3.2885 ± 0.07293	297.22 ± 5.5367	2.9789 ± 0.02461^b^
		TT (460)	9,970.53 ± 177.07	322.44 ± 7.5119^B^	3.2569 ± 0.072	295.75 ± 5.4744	2.9785 ± 0.02422^b^
		*p*	0.6498	0.0308	0.2369	0.1995	0.0313
	2	CC (43)	11507 ± 239.38^A^	420.86 ± 10.0507^A^	3.6252 ± 0.09705	332.26 ± 7.3264^a^	2.8773 ± 0.03295^A^
		CT (270)	11115 ± 221.72^B^	413.29 ± 9.437^A^	3.6831 ± 0.09033	325.59 ± 6.8773^b^	2.93 ± 0.02998^B^
		TT (320)	11064 ± 218.52^B^	406.53 ± 9.3118^B^	3.6511 ± 0.08905	325.06 ± 6.7858^b^	2.9392 ± 0.02954^B^
		*p*	0.001	0.0007	0.2554	0.0992	0.0023
3:g.15344349A>C	1	AA (67)	9,991.39 ± 192.06	325.46 ± 8.0348^a^	3.2756 ± 0.07773	299.91 ± 5.8571	3.0135 ± 0.02668^Aa^
		AC (397)	10005 ± 179.51	326.98 ± 7.5972^ab^	3.2894 ± 0.07293	297.03 ± 5.5368	2.9797 ± 0.02461^ABb^
		CC (461)	9,974.55 ± 177.08	322.54 ± 7.512^b^	3.2564 ± 0.072	295.84 ± 5.4744	2.9781 ± 0.02423^aBb^
		*p*	0.8101	0.0497	0.2086	0.248	0.0306
	2	AA (43)	11517 ± 239.39^A^	421.11 ± 10.051^A^	3.6242 ± 0.09705	332.59 ± 7.3267^a^	2.8775 ± 0.03295^A^
		AC (268)	11148 ± 221.77^B^	414.03 ± 9.4388^A^	3.4796 ± 0.09035	326.61 ± 6.8786^ab^	2.9306 ± 0.02999^B^
		CC (322)	11050 ± 218.52^B^	406.27 ± 9.3117^B^	3.6527 ± 0.08905	324.67 ± 6.7858^b^	2.9389 ± 0.02954^B^
		*p*	0.0004	0.0002	0.3358	0.0539	0.0026
3:g.15345216C>T	1	CC (67)	9,991.39 ± 192.06	325.46 ± 8.0348^ab^	3.2756 ± 0.07773	299.91 ± 5.8571	3.0135 ± 0.02668^Aa^
		CT (397)	10005 ± 179.51	326.98 ± 7.5972^a^	3.2894 ± 0.07293	297.03 ± 5.5368	2.9797 ± 0.02461^ABb^
		TT (461)	9,974.55 ± 177.08	322.54 ± 7.512^b^	3.2564 ± 0.072	295.84 ± 5.4744	2.9781 ± 0.02423^aBb^
		*p*	0.8101	0.0497	0.2086	0.248	0.0306
	2	CC (43)	11517 ± 239.39^A^	421.11 ± 10.051^A^	3.6242 ± 0.09705	332.59 ± 7.3267^a^	2.8775 ± 0.03295^A^
		CT (268)	11148 ± 221.77^B^	414.03 ± 9.4388^A^	3.6796 ± 0.09035	326.61 ± 6.8786^ab^	2.9306 ± 0.02999^B^
		TT (322)	11050 ± 218.52^B^	406.27 ± 9.3117^B^	3.6527 ± 0.08905	324.67 ± 6.7858^b^	2.9389 ± 0.02954^B^
		*p*	0.0004	0.0002	0.3358	0.0539	0.0026
3:g.15345227T>C	1	CC (462)	9,976.27 ± 177.07	322.64 ± 7.5119^a^	3.2568 ± 0.072	295.89 ± 5.4744	2.9781 ± 0.02422^Aab^
		CT (396)	10001 ± 179.52	326.76 ± 7.5976^b^	3.2888 ± 0.07294	296.91 ± 5.5371	2.9797 ± 0.02462^AaB^
		TT (67)	9,990.25 ± 192.06	325.4 ± 8.0349^ab^	3.2754 ± 0.07773	299.87 ± 5.8572	3.0135 ± 0.02668^Bb^
		*p*	0.8681	0.0746	0.2289	0.2745	0.0306
	2	CC (323)	11052 ± 218.52^A^	406.42 ± 9.3116^A^	3.6535 ± 0.08905	324.72 ± 6.7857^a^	2.9388 ± 0.02953^A^
		CT (267)	11144 ± 221.8^A^	413.65 ± 9.4399^B^	3.6778 ± 0.09036	326.5 ± 6.8794^ab^	2.9307 ± 0.03^A^
		TT (43)	11516 ± 239.39^B^	420.99 ± 10.0512^B^	3.6236 ± 0.09705	332.56 ± 7.3268^b^	2.8775 ± 0.03295^B^
		*p*	0.0005	0.0004	0.3808	0.0596	0.0026
3:g.15349740A>G	1	AA (66)	9,984.83 ± 192.32	324.8 ± 8.0442^ab^	3.271 ± 0.07783	299.64 ± 5.864	3.0128 ± 0.02672^a^
		AG (398)	10007 ± 179.5	327.15 ± 7.5969^a^	3.2906 ± 0.07293	297.11 ± 5.5366	2.98 ± 0.02461^b^
		GG (461)	9,975.25 ± 177.08	322.61 ± 7.5122^b^	3.2569 ± 0.07201	295.86 ± 5.4746	2.9781 ± 0.02423^b^
		*p*	0.7958	0.0436	0.1938	0.2894	0.0383
	2	AA (42)	11509 ± 239.99^A^	422.11 ± 10.0716^A^	3.6379 ± 0.09728	332.38 ± 7.3417^a^	2.8778 ± 0.03305^A^
		AG (269)	11152 ± 221.74^B^	413.8 ± 9.4379^A^	3.6757 ± 0.09034	326.7 ± 6.8779^ab^	2.9302 ± 0.02999^B^
		GG (322)	11052 ± 218.52^B^	406.17 ± 9.3116^B^	3.651 ± 0.08905	324.71 ± 6.7857^b^	2.9387 ± 0.02953^B^
		*p*	0.0006	0.0001	0.4922	0.0649	0.0031
3:g.15349768A>G	1	AA (66)	9,984.83 ± 192.32	324.8 ± 8.0442^ab^	3.271 ± 0.07783	299.64 ± 5.864	3.0128 ± 0.02672^a^
		AG (398)	10007 ± 179.5	327.15 ± 7.5969^a^	3.2906 ± 0.07293	297.11 ± 5.5366	2.98 ± 0.02461^b^
		GG (461)	9,975.25 ± 177.08	322.61 ± 7.5122^b^	3.2569 ± 0.07201	295.86 ± 5.4746	2.9781 ± 0.02423^b^
		*p*	0.7958	0.0436	0.1938	0.2894	0.0383
	2	AA (42)	11509 ± 239.99^A^	422.11 ± 10.0716^A^	3.6379 ± 0.09728	332.38 ± 7.3417^a^	2.8778 ± 0.03305^A^
		AG (269)	11152 ± 221.74^B^	413.8 ± 9.4379^A^	3.6757 ± 0.09034	326.7 ± 6.8779^ab^	2.9302 ± 0.02999^B^
		GG (322)	11052 ± 218.52^B^	406.17 ± 9.3116^B^	3.651 ± 0.08905	324.71 ± 6.7857^b^	2.9387 ± 0.02953^B^
		*p*	0.0006	0.0001	0.4922	0.0649	0.0031
3:g.15349978A>G	1	AA (66)	9,983.68 ± 192.33	324.74 ± 8.0443^ab^	3.2708 ± 0.07783	299.6 ± 5.8641	3.0128 ± 0.02672^a^
		AG (397)	10003 ± 179.51	326.94 ± 7.5972^a^	3.29 ± 0.07293	297 ± 5.5368	2.98 ± 0.02461^b^
		GG (462)	9,976.97 ± 177.08	322.71 ± 7.5121^b^	3.2573 ± 0.07201	295.91 ± 5.4745	2.9781 ± 0.02423^b^
		*p*	0.8549	0.0661	0.213	0.322	0.0383
	2	AA (42)	11508 ± 239.99^A^	421.99 ± 10.0718^A^	3.6373 ± 0.09782	332.34 ± 7.3419^a^	2.8778 ± 0.03305^A^
		AG (268)	11148 ± 221.77^B^	413.42 ± 9.439^A^	3.6739 ± 0.09035	326.59 ± 6.8787^ab^	2.9303 ± 0.02999^B^
		GG (323)	11053 ± 218.51^B^	406.32 ± 9.3115^B^	3.6518 ± 0.08905	324.75 ± 6.7856^b^	2.9387 ± 0.02953^B^
		*p*	0.0007	0.0003	0.5521	0.072	0.0032
3:g.15350548C>T	1	CC (66)	9,984.83 ± 192.32	324.8 ± 8.0442^ab^	3.271 ± 0.07783	299.64 ± 5.864	3.0128 ± 0.02672^a^
		CT (398)	10007 ± 179.5	327.15 ± 7.5969^a^	3.2906 ± 0.07293	297.11 ± 5.5366	2.98 ± 0.02461^b^
		TT (461)	9,975.25 ± 177.08	322.61 ± 7.5122^b^	3.2569 ± 0.07201	295.86 ± 5.4746	2.9781 ± 0.02423^b^
		*p*	0.7958	0.0436	0.1938	0.2894	0.0383
	2	CC (42)	11509 ± 239.99^A^	422.11 ± 10.0716^A^	3.6379 ± 0.09728	332.38 ± 7.3417^a^	2.8778 ± 0.03305^A^
		CT (269)	11152 ± 221.74^B^	413.8 ± 9.4379^A^	3.6757 ± 0.09034	326.7 ± 6.8779^ab^	2.9302 ± 0.02999^B^
		TT (322)	11052 ± 218.52^B^	406.17 ± 9.3116^B^	3.651 ± 0.08905	324.71 ± 6.7857^b^	2.9387 ± 0.02953^B^
		*p*	0.0006	0.0001	0.4922	0.0649	0.0031
3:g.15350655A>G	1	AA (66)	9,984.83 ± 192.32	324.8 ± 8.0442^ab^	3.271 ± 0.07783	299.64 ± 5.864	3.0128 ± 0.02672^a^
		AG (398)	10007 ± 179.5	327.15 ± 7.5969^a^	3.2906 ± 0.07293	297.11 ± 5.5366	2.98 ± 0.02461^b^
		GG (461)	9,975.25 ± 177.08	322.61 ± 7.5122^b^	3.2569 ± 0.07201	295.86 ± 5.4746	2.9781 ± 0.02423^b^
		*p*	0.7958	0.0436	0.1938	0.2894	0.0383
	2	AA (42)	11509 ± 239.99^A^	422.11 ± 10.0716^A^	3.6379 ± 0.09728	332.38 ± 7.3417^a^	2.8778 ± 0.03305^A^
		AG (269)	11152 ± 221.74^B^	413.8 ± 9.4379^A^	3.6757 ± 0.09034	326.7 ± 6.8779^ab^	2.9302 ± 0.02999^B^
		GG (322)	11052 ± 218.52^B^	406.17 ± 9.3116^B^	3.651 ± 0.08905	324.71 ± 6.7857^b^	2.9387 ± 0.02953^B^
		*p*	0.0006	0.0001	0.4922	0.0649	0.0031
3:g.15350805T>C	1	CC (461)	9,975.25 ± 177.08	322.61 ± 7.5122^a^	3.2569 ± 0.07201	295.86 ± 5.4746	2.9781 ± 0.02423^a^
		CT (398)	10007 ± 179.5	327.15 ± 7.5969^b^	3.2906 ± 0.07293	297.11 ± 5.5366	2.98 ± 0.02461^a^
		TT (66)	9,984.83 ± 192.32	324.8 ± 8.0442^ab^	3.271 ± 0.07783	299.64 ± 5.864	3.0128 ± 0.02672^b^
		*p*	0.7958	0.0436	0.1938	0.2894	0.0383
	2	CC (322)	11052 ± 218.52^A^	406.17 ± 9.3116^A^	3.651 ± 0.08905	324.71 ± 6.7857^a^	2.9387 ± 0.02953^A^
		CT (269)	11152 ± 221.74^A^	413.8 ± 9.4379^B^	3.6757 ± 0.09034	326.7 ± 6.8779^ab^	2.9302 ± 0.02999^A^
		TT (42)	11509 ± 239.99^B^	422.11 ± 10.0716^B^	3.6379 ± 0.09728	332.38 ± 7.3417^b^	2.8778 ± 0.03305^B^
		*p*	0.0006	0.0001	0.4922	0.0649	0.0031
3:g.15350898T>C	1	CC (26)	9,605.25 ± 218.94^Aab^	310.38 ± 8.9936^Aa^	3.2436 ± 0.08808	284.92 ± 6.5587^A^	2.9832 ± 0.03096^ab^
		CT (286)	10067 ± 179.53^aB^	326.76 ± 7.5947^aBb^	3.2677 ± 0.07293	297.89 ± 5.535^B^	2.9717 ± 0.02464^a^
		TT (613)	9,958.02 ± 177.04^Bb^	323.02 ± 7.5115^ABb^	3.2673 ± 0.07199	296.45 ± 5.4741^B^	2.9886 ± 0.02421^b^
		*p*	0.0016	0.0042	0.9024	0.0035	0.0981
	2	CC (18)	11103 ± 279.57	414.38 ± 11.5034	3.7121 ± 0.1126	328.79 ± 8.3889	2.9547 ± 0.03933^ab^
		CT (189)	11144 ± 220.3	407.97 ± 9.371	3.6433 ± 0.08972	325.12 ± 6.8292	2.917 ± 0.02985^a^
		TT (426)	11126 ± 219.59	411.38 ± 9.3557	3.6621 ± 0.08949	326.94 ± 6.8179	2.9373 ± 0.02967^b^
		*p*	0.9493	0.34	0.5521	0.548	0.0781
3:g.15352855T>C	1	CC (461)	9,970.2 ± 177.08	322.5 ± 7.512^A^	3.2577 ± 0.072	295.73 ± 5.4745	2.9783 ± 0.02423^a^
		CT (398)	10018 ± 179.5	327.41 ± 7.5968^B^	3.2889 ± 0.07293	297.41 ± 5.5365	2.9795 ± 0.02461^a^
		TT (66)	9,988.28 ± 192.32	324.87 ± 8.044^AB^	3.2704 ± 0.07783	299.73 ± 5.8639	3.0126 ± 0.02672^b^
		*p*	0.5913	0.0258	0.2449	0.2005	0.0391
	2	CC (321)	11060 ± 218.51^A^	406.48 ± 9.3115^A^	3.6516 ± 0.08905	325.01 ± 6.7856^a^	2.9396 ± 0.02953^A^
		CT (270)	11131 ± 221.71^A^	412.95 ± 9.4368^B^	3.6742 ± 0.09032	325.91 ± 6.8771^ab^	2.9285 ± 0.02998^A^
		TT (42)	11503 ± 239.98^B^	421.83 ± 10.0714^B^	3.6374 ± 0.09728	332.12 ± 7.3416^b^	2.8773 ± 0.03305^B^
		*p*	0.0012	0.0006	0.5375	0.1129	0.0023
3:g.15353088A>C	1	AA (66)	9,986.16 ± 192.32	324.85 ± 8.0442^AB^	3.271 ± 0.07783	299.67 ± 5.864	3.0127 ± 0.02672^a^
		AC (399)	10011 ± 179.49	327.31 ± 7.5967^A^	3.2906 ± 0.07293	297.22 ± 5.5365	2.9798 ± 0.02461^b^
		CC (460)	9,973.32 ± 177.08	322.54 ± 7.5122^B^	3.2569 ± 0.07201	295.81 ± 5.4746	2.9782 ± 0.02423^b^
		*p*	0.7219	0.0317	0.1945	0.2565	0.0387
	2	AA (42)	11503 ± 239.98^A^	421.83 ± 10.0714^A^	3.6374 ± 0.09728	332.12 ± 7.3416^a^	2.8773 ± 0.03305^A^
		AC (270)	11131 ± 221.71^B^	412.95 ± 9.4368^A^	3.6742 ± 0.09032	325.91 ± 6.8771^ab^	2.9285 ± 0.02998^B^
		CC (321)	11060 ± 218.51^B^	406.48 ± 9.3115^B^	3.6516 ± 0.08905	325.01 ± 6.7856^b^	2.9396 ± 0.02953^B^
		*p*	0.0012	0.0006	0.5375	0.1129	0.0023
3:g.15353235T>C	1	CC (458)	9,975.3 ± 177.07	322.62 ± 7.5117^A^	3.2571 ± 0.072	295.91 ± 5.4742	2.9786 ± 0.02422^Aab^
		CT (400)	10020 ± 179.44	327.58 ± 7.5948^B^	3.2902 ± 0.07291	297.41 ± 5.535	2.9789 ± 0.02461^AaB^
		TT (67)	9,958.69 ± 192.25	323.73 ± 8.042^AB^	3.2698 ± 0.0778	298.9 ± 5.8624	3.0139 ± 0.02671^Bb^
		*p*	0.5843	0.0224	0.2057	0.3534	0.0279
	2	CC(320)	110073 ± 218.46^A^	406.61 ± 9.309^A^	3.6482 ± 0.08903	325.26 ± 6.7838	2.9382 ± 0.02953^A^
		CT (270)	11132 ± 221.57^A^	413.3 ± 9.4313^B^	3.6768 ± 0.09027	325.94 ± 6.8731	2.9284 ± 0.02996^B^
		TT (43)	11463 ± 239.82^B^	421.26 ± 10.0661^B^	3.6472 ± 0.09722	331.25 ± 7.3377	2.8812 ± 0.03302^B^
		*p*	0.0046	0.0006	0.4487	0.2026	0.0054
3:g.15353254T>C	1	CC (458)	9,975.3 ± 177.07	322.62 ± 7.5117^A^	3.2571 ± 0.072	295.91 ± 5.4742	2.9786 ± 0.02422^Aab^
		CT (400)	10020 ± 179.44	327.58 ± 7.5948^B^	3.2902 ± 0.07291	297.41 ± 5.535	2.9789 ± 0.02461^AaB^
		TT (67)	9,958.69 ± 192.25	323.73 ± 8.042^AB^	3.2698 ± 0.0778	298.9 ± 5.8624	3.0139 ± 0.02671^Bb^
		*p*	0.5843	0.0224	0.2057	0.3534	0.0279
	2	CC (320)	11073 ± 218.46^A^	406.61 ± 9.309^A^	3.6482 ± 0.08903	325.26 ± 6.7838	2.9382 ± 0.02953^A^
		CT (270)	11132 ± 221.57^A^	413.3 ± 9.4313^B^	3.6768 ± 0.09027	325.94 ± 6.8731	2.9284 ± 0.02996^A^
		TT (43)	11463 ± 239.82^B^	421.26 ± 10.0661^B^	3.6472 ± 0.09722	331.25 ± 7.3377	2.8812 ± 0.03302^B^
		*p*	0.0046	0.0006	0.4487	0.2026	0.0054
3:g.15353292C>G	1	CC (70)	9,944.8 ± 191.52	322.37 ± 8.016^AB^	3.2614 ± 0.07752	298.06 ± 5.8434	3.01 ± 0.02659^a^
		CG (398)	10030 ± 179.48	328.03 ± 7.5959^A^	3.2913 ± 0.07292	297.73 ± 5.5359	2.9791 ± 0.02461^b^
		GG (457)	9,974.5 ± 177.06	322.75 ± 7.5113^B^	3.2585 ± 0.072	295.96 ± 5.4739	2.9792 ± 0.02422^b^
		*p*	0.4024	0.0097	0.1968	0.3649	0.0565
	2	CC (45)	11416 ± 238.71^Aa^	419.51 ± 10.0282^A^	3.6449 ± 0.0968	330.41 ± 7.31	2.8865 ± 0.03283^Aa^
		CG (268)	11140 ± 221.63^ABb^	413.68 ± 9.4334^A^	3.6778 ± 0.09029	326.11 ± 6.8746	2.9276 ± 0.02997^ABb^
		GG (320)	11077 ± 218.46^aBb^	406.77 ± 9.3091^B^	3.6485 ± 0.08903	325.33 ± 6.7838	2.9379 ± 0.02953^aBb^
		*p*	0.0135	0.0012	0.4243	0.302	0.0114
3:g.15353330A>G	1	AA (79)	9,899.56 ± 189.6	322.46 ± 7.9485^AB^	3.2788 ± 0.07679	297 ± 5.794	3.013 ± 0.02628^A^
		AG (389)	10046 ± 179.54	328.15 ± 7.5982^A^	3.2868 ± 0.07295	298.05 ± 5.5376	2.9774 ± 0.02462^B^
		GG (457)	9,979.48 ± 177.05	322.76 ± 7.511^B^	3.2568 ± 0.07199	296.07 ± 5.4737	2.9787 ± 0.02422^B^
		*p*	0.1389	0.0081	0.276	0.3331	0.0153
	2	AA (50)	11426 ± 236.22^A^	421.24 ± 9.9409^A^	3.6591 ± 0.09585	331.51 ± 7.2461^a^	2.8942 ± 0.03242^Aa^
		AG (263)	11129 ± 221.8^B^	412.97 ± 9.4393^A^	3.6745 ± 0.09036	325.64 ± 6.879^ab^	2.9267 ± 0.03^ABb^
		GG (320)	11074 ± 218.47^B^	406.52 ± 9.3093^B^	3.6473 ± 0.08903	325.18 ± 6.784^b^	2.9375 ± 0.02953^aBb^
		*p*	0.0063	0.0005	0.5163	0.1237	0.0275
3:g.15353342C>T	1	CC (82)	9,920.86 ± 189.11	321.48 ± 7.9312^AaB^	3.2598 ± 0.0766	297.12 ± 5.7813	3.0075 ± 0.0262^a^
		CT (387)	10045 ± 179.6	328.74 ± 7.6001^Ab^	3.2934 ± 0.07297	298.09 ± 5.5389	2.9783 ± 0.02463^b^
		TT (456)	9,975.84 ± 177.05	322.8 ± 7.5109^aBb^	3.2587 ± 0.07199	296.03 ± 5.4736	2.9795 ± 0.02422^b^
		*p*	0.1847	0.002	0.1603	0.3057	0.0546
	2	CC (50)	11394 ± 236.17^Aa^	417.79 ± 9.9384^AaB^	3.6365 ± 0.09582	330.51 ± 7.2443	2.8945 ± 0.03241^A^
		CT (263)	11138 ± 221.8^ABb^	414.03 ± 9.4395^Aab^	3.6814 ± 0.09036	325.94 ± 6.8791	2.9266 ± 0.03^AB^
		TT (320)	11077 ± 218.46^aBb^	406.92 ± 9.3091^Bb^	3.6496 ± 0.08903	325.3 ± 6.7838	2.9374 ± 0.02953^B^
		*p*	0.0157	0.0019	0.3239	0.2462	0.0285
3:g.15355389T>C	1	CC (455)	9,959.33 ± 176.95^ab^	321.99 ± 7.5076^Aab^	3.257 ± 0.07196	295.75 ± 5.4712^ab^	2.9821 ± 0.0242
		CT (358)	10028 ± 179.57^a^	327.84 ± 7.5987^aB^	3.2897 ± 0.07295	298.36 ± 5.5379^a^	2.9864 ± 0.02463
		TT (109)	9,872.62 ± 186.83^b^	321.17 ± 7.854^ABb^	3.2816 ± 0.07574	293.97 ± 5.7248^b^	2.9925 ± 0.02581
		*p*	<0.0001	<0.0001	0.3159	0.0002	0.0374
	2	CC (317)	11088 ± 218.46	408.02 ± 9.3087^A^	3.656 ± 0.08903^ab^	325.66 ± 6.7835	2.9377 ± 0.02953^A^
		CT (241)	11207 ± 222.08	417.82 ± 9.4492^B^	3.6914 ± 0.09046^a^	327.92 ± 6.8862	2.926 ± 0.03005^AB^
		TT (71)	11213 ± 231.31	407.7 ± 9.7655^A^	3.6031 ± 0.09396^b^	325.7 ± 7.1178	2.8987 ± 0.03161^B^
		*p*	0.2155	0.0003	0.1161	0.4657	0.0585
3:g.15355514T>C	1	CC (457)	9,985.46 ± 176.88^ab^	322.9 ± 7.5049^A^	3.2557 ± 0.07193	296.38 ± 5.4692^ab^	2.9799 ± 0.0242
		CT (358)	10045 ± 179.49^a^	328.34 ± 7.5957^B^	3.2879 ± 0.07292	298.76 ± 5.5357^a^	2.9847 ± 0.02462
		TT (108)	9,866.46 ± 187.14^b^	320.88 ± 7.8657^A^	3.2799 ± 0.07586	293.88 ± 5.7333^b^	2.9928 ± 0.02586
		*p*	0.0004	0.0003	0.3029	0.0044	0.0152
	2	CC (318)	11079 ± 218.46^a^	407.65 ± 9.3088^Aab^	3.6551 ± 0.08903^ab^	325.34 ± 6.7836^a^	2.9368 ± 0.02953^a^
		CT (240)	11229 ± 222.11^b^	418.7 ± 9.4505^aB^	3.6928 ± 0.09048^a^	328.76 ± 6.8872^b^	2.9277 ± 0.03005^ab^
		TT (72)	11227 ± 231.13^ab^	409.02 ± 9.7594^ABb^	3.6098 ± 0.09389^b^	326.41 ± 7.1133^ab^	2.9012 ± 0.03158^b^
		*p*	0.0866	<0.0001	0.0542	0.2667	0.0883
3:g.15355833A>G	1	AA (107)	9,845.22 ± 187.08^a^	320.15 ± 7.8632^AaB^	3.2817 ± 0.07583	293.31 ± 5.7315^a^	2.9945 ± 0.02585
		AG (358)	10007 ± 179.62^b^	327.02 ± 7.601^Ab^	3.2898 ± 0.07298	297.7 ± 5.5396^b^	2.9868 ± 0.02464
		GG (458)	9,951 ± 176.98^ab^	321.72 ± 7.509^aBb^	3.2577 ± 0.07197	295.45 ± 5.4722^ab^	2.9822 ± 0.02421
		*p*	<0.0001	<0.0001	0.3381	<0.0001	0.0743
	2	AA (71)	11216 ± 231.27^ab^	408.03 ± 9.7644^A^	3.6059 ± 0.09395^a^	325.9 ± 7.117	2.8997 ± 0.03161^a^
		AG (240)	11229 ± 222.11^a^	418.77 ± 9.4505^B^	3.6936 ± 0.09048^b^	328.77 ± 6.8871	2.9279 ± 0.03005^ab^
		GG (319)	11082 ± 218.45^b^	407.55 ± 9.3085^A^	3.6525 ± 0.08902^ab^	325.37 ± 6.7834	2.936 ± 0.02953^b^
		*p*	0.1047	<0.0001	0.0553	0.1983	0.0436

Note: LSM ±SE: Least Squares Mean ± Standard Deviation; the number in the bracket represents the number of cows for the corresponding genotype; *p* shows the significance for the genetic effects of SNPs; a, b within the same column with different superscripts means *p* < 0.05; and A, B within the same column with different superscripts means *p* < 0.01.

### Associations between haplotype block and five milk productions traits

**FIGURE 1:**
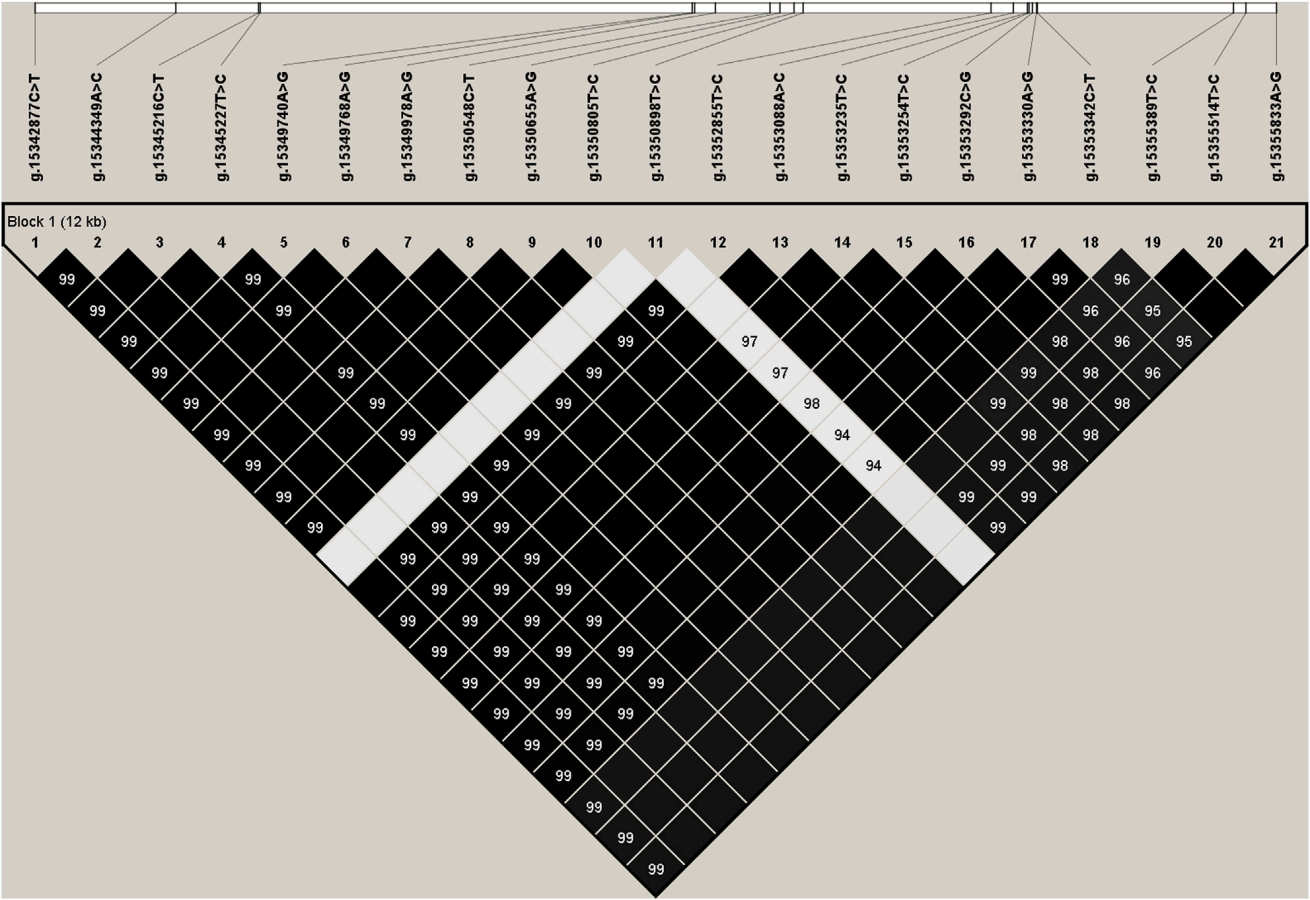
Linkage disequilibrium estimated between SNPs in *PKLR* gene. The values in the black boxes are pair-wise SNP correlations (*R* ^2^).

We estimated the degree of linkage disequilibrium (LD) among the 21 identified SNPs in *PKLR* gene using Haploview4.2, and inferred one haplotype block including all the SNPs (Figure 1). The block consisted of four haplotypes, H1 (TCT​CGG​GTG​CTC​CCC​GGT​CCG), H2 (CAC​TAA​ACA​TTT​ATT​CAC​TTA), H3 (TCT​CGG​GTG​CCC​CCC​GGT​CCG), and H4 (TCT​CGG​GTG​CTC​CCC​GGT​TTA) with the frequencies of 0.499, 0.287, 0.181, and 0.021, respectively. The haplotype combinations demonstrated significant associations with fat yield and protein percentage in the first and second lactations (*p* ≤ 0.0145), and milk yield (*p* = 0.0003) and protein yield (*p* = 0.0183) in the second lactation (Supplementary Table S5).

### Regulation of the 5′ region SNPs on transcriptional activity

We used the MEME Suite software to predict the changes of TFBSs caused by the four SNPs on the 5′ regulatory region of *PKLR* gene. The detailed results were shown in Table 3. The allele C of 3:g.15342877C>T created binding sites (BSs) for transcription factors (TFs) SP100 and ESRRA. In 3:g.15344349A>C, allele A created BSs for three TFs, MLX, ZBTB33 and IRF5, and the allele C created the BSs for ZNF524, YY2, and SREBF2. As for 3:g.15345216C>T, the allele C invented BS for RREB1, the allele T invented BSs for TWIST2, ZEB1, NAC007, BHLHE22, ZFP42, TCF3, NAC031, ZSCAN31, and TCF12. The allele C of 3:g.15345227T>C created BSs for TFs MYC, TFAP2A and TCF4.

**TABLE 3:** Changes in transcription factors binding sites (TFBSs) caused by the SNPs in 5′ regulatory region of *PKLR*.

SNP name	Allele	TFs	*p*	Predicted core binding site sequence
3:g.15342877C>T	C	SP100	0.0030	TCCGTCGCTTAAAAG
		ESRRA	0.0046	TAGGTCAGTCAAGGTCA
	T	—	—	—
3:g.15344349A>C	A	MLX	0.0034	ATCACGTGAT
		ZBTB33	0.0042	CTCTCGCGAGATCTG
		IRF5	0.0048	TTGATCGAGAATTCC
	C	ZNF524	0.0014	ACCCTCGAACCC
		YY2	0.0021	CCATGCCGCCAT
		SREBF2	0.0044	ATCACGTGAC
3:g.15345216C>T	C	RREB1	0.0031	CCC​CAA​ACC​ACCCCCC​CCC​C
	T	TWIST2	0.0008	CGCAGCTGCG
		ZEB1	0.0009	CCCACCTGCGC
		NAC007	0.0010	GCCAGCTGGC
		BHLHE22	0.0016	CGCAGCTGCG
		ZFP42	0.0016	GTT​CCA​AAATGGC​TGC​CTC​CG
		TCF3	0.0024	CGCACCTGCCC
		NAC031	0.0029	AGCAGCTGCT
		ZSCAN31	0.0030	GCA​TAA​CTGCC​CTG​CGT​CC
		TCF12	0.0046	CGCACCTGCCG
3:g.15345227T>C	T	—	—	—
	C	MYC	0.0029	GGCCACGTGCCC
		TFAP2A	0.0031	ATTGCCTCAGGCCA
		TCF4	0.0032	CGGCACCTGCCCC

Note: TFs: transcription factors; SNP, site is underlined.

### Prediction of changes in secondary structures of mRNA

We used the RNAfold Web Server to predict the changes of secondary structures of mRNA for thirteen SNPs in UTR and exon regions of *PKLR* gene. All the thirteen SNP mutation sites were predicted to change the MFE of mRNA secondary structures compared to the MFE of reference sequence (XM_024989616.1; ARS-UCD1.2; Table 4). Among them, six sites, 3:g.15345216T, 3:g.15345227C, 3:g.15349768G, 3:g.15350898C, 3:g.15353235C and 3:g.15353254C, could increase the MFE to cause the instability of *PKLR* mRNA secondary structure, and the other seven sites, 3:g.15349978G, 3:g.15350655G, 3:g.15352855C, 3:g.15353088C, 3:g.15353292G, 3:g.15353330G, and 3:g.15353342T, could decrease the MFE and make the mRNA secondary structure more stable*.*

**TABLE 4:** The minimum free energy (MFE) values of optimal secondary structure of *PKLR* mRNA.

Mutant site	MFE (kcal/mol)
References sequence	−1,145.2
3:g.15345216T	−1,143
3:g.15345227C	−1,144.9
3:g.15349768G	−1,144.5
3:g.15349978G	−1,146
3:g.15350655G	−1,148.80
3:g.15350898C	−1,143.3
3:g.15352855C	−1,145.6
3:g.15353088C	−1,147.20
3:g.15353235C	−1,144.6
3:g.15353254C	−1,143.90
3:g.15353292G	−1,149.3
3:g.15353330G	−1,151.80
3:g.15353342T	−1,150

Note: MFE: minimum free energy; reference sequence: XM_024989616.1 (ARS-UCD1.2).

## Discussion

Our previous study considered *PKLR* gene to be a candidate to affect milk production traits in dairy cattle (Liang et al., 2017). In this study, we identified totally 21 SNPs in *PKLR* gene, and found that all the SNPs were significantly associated with at least one milk production trait, simultaneously, the results of haplotype association analysis were basically consistent with the single marker association analysis, which suggested that the *PKLR* gene had large genetic effect on milk production traits. Brondum et al. (2015) added the sequence data of a few significant variation into the conventional 54k SNPs for single marker analysis, and found it can improve the reliability of genomic prediction, for instance, the reliability of the Nordic Holstein cattle milk production traits increased by 4%, that of Nordic red bull increased by 3%, and that of France Holstein cows increased by 5%. Currently, four commercial gene chips, including illumina Bovine SNP50K BeadChip, illumina BovineHD Genotyping BeadChip, GeneSeek Genomic Profiler (GGP) Bovine 150K, and 100K arrays, do not contain SNPs identified in this study, after that, we could try to add significant functional SNPs in this study to gene chips to improve the accuracy of genomic prediction in dairy cattle.

*PKLR* converts phosphoenolpyruvic acid to pyruvate, the main carbon source, and its perturbation may significantly affect the pyruvate levels in cells (Liu et al., 2019). Moreover, pyruvate is an important intermediate in the glucose metabolism of all living organisms and the mutual transformation of various substances in the body. It can also convert sugars, fats and amino acids into each other through acetyl CoA and the tricarboxylic acid cycle (Gray et al., 2014). Studies have shown that *PKLR* regulates and influences key metabolic pathways related to lipid metabolism, steroid biosynthesis, PPAR signaling pathway, fatty acid synthesis and oxidation (Lee et al., 2017; Mardinoglu et al., 2018; Liu et al., 2019). It can be seen that *PKLR* gene can regulate the synthesis of milk components, especially milk lactose and fat.

Transcription factors are a group of protein molecules that bind to TFBSs to ensure that the target gene is expressed at a specific intensity at a specific time and space (Jolma et al., 2013). When the mutation site changes that it will affect the binding of TFs to TFBSs, and then inhibiting or enhancing gene expression (Spivakov et al., 2012). In this study, four SNPs in 5′ region of *PKLR* were predicted to change the TFBSs that would be affect the expression of the downstream gene. For the 3:g.15342877C>T, the allele C could bind SP100 and ESRRA, and the milk and fat yields of CC genotype cows was significantly higher than that of TT individuals. In addition, it has reported that ESRRA enhanced the transcriptional activation of numerous autophagy-related (Atg) genes, *Atg5*, *Atg16l1*, and *Becn1* (Kim et al., 2018). SP100 may function as a nuclear hormone receptor transcriptional coactivator (Bloch et al., 2000). It can be inferred that the increase of CC genotype phenotype may be due to the combination of transcription factors SP100 and ESRRA at the C site, which together activate the expression of gene *PKLR*. The allele A in 3:g.15344349A>C could bind MLX, ZBTB33, and IRF5, and the allele C binds ZNF524, YY2, and SREBF2, meanwhile, the milk and fat yields of AA genotype cows was significantly higher than that of CC individuals. MLX plays a role in transcriptional activation of glycolytic target genes and the Mondo family (Billin et al., 2000; Sans et al., 2006). ZBTB33 activated transcription from exogenous methylated promoters (Zhenilo et al., 2018). IRF5 directly activated transcription of the genes *IL-12p40*, *IL-12p35,* and *IL-23p19* and contributed to the plasticity of macrophage polarization (Krausgruber et al., 2011). YY2 reduces the activity of the *c-Myc* and *CXCR4* promoter (Nguyen et al., 2004). SREBF2 can activate the transcription of genes involved in cholesterol biosynthesis (Xu et al., 2020; Sellers et al., 2021). The functional role of TF ZNF524 is unclear so far. It is speculated that the higher milk yield of AA genotype cows may be the result of combined activation of transcription factors MLX, ZBTB33 and IRF5 or the inhibition of ZNF524, YY2, and SREBF2 on the expression of gene *PKLR*. For the 3:g.15345216C>T, the allele C binds RREB1, and allele T could bind TWIST2, ZEB1, NAC007, BHLHE22, ZFP42, TCF3, NAC031, ZSCAN31, and TCF12, as well as, the milk and fat yields of CC genotype cows was significantly higher than that of TT individuals. RREB1 is a transcriptional activator of calcitonin in response to Ras signaling (Deng et al., 2020). TWIST2 can suppress the expression of *FGF21* to activate the AMPK/mTOR signalling pathway which inhibits the progression of various cancers (Song et al., 2021). ZEB1 as a direct transcriptional repressor of E-cadherin by physically binding to the proximal promoter of E-cadherin in breast cancers (Eger et al., 2005). BHLHE22 is a transcriptional repressor and is involved in cell differentiation in neuron development (Ross et al., 2012; Darmawi et al., 2022), TCF3 combined with HDAC3 down-regulates the expression of miR-101 that is a type of tumor suppressor gene, thereby promoting the proliferation of BL cells and inhibiting their apoptosis (Dong et al., 2021). TCF12 functions as transcriptional repressor of E-cadherin (Lee et al., 2012). The function of some transcription factors, NAC007, ZFP42, NAC031, and ZSCAN31, is still unclear. Therefore, it can be speculated that the increased phenotype of CC genotype individuals may be caused by the activation of *PKLR* gene expression by binding the TF RREB1, or the co-inhibition of *PKLR* gene expression by TFs TWIST2, ZEB1, NAC007, BHLHE22, ZFP42, TCF3, NAC031, ZSCAN31, and TCF12. For the 3:g.15345227T>C, the allele C could bind MYC, TFAP2A and TCF4, and the milk and fat yields of CC genotype cows was significantly lower than that of TT individuals. MYC represses transcription when tethered to promoters by Miz1 or other proteins (Adhikary and Eilers 2005). TFAP2A appeared to strengthen the binding of Smad2/3 to target promoters and affect transcriptional responses in knockdown experiments (Koinuma et al., 2009). TCF4 is involved in the initiation of neuronal differentiation by binding to the E box to activate transcription (Teixeira et al., 2021). It can be speculated that the decrease of CC genotype phenotype may be due to the combination of TFs MYC, TFAP2A, and TCF4 to inhibit the expression of *PKLR* gene. Thus, we speculated that these four SNP mutations changed the TFBSs to modulate the gene expression of *PKLR*, resulting in changes of phenotypic data.

The secondary structure of mRNA is formed by the complementary pairing of bases on the single chain, and the same mRNA molecules can be folded to form a variety of configurations. The secondary structure of mRNA, as the skeleton of the higher functions of RNA, plays an important role in various life processes, including protein folding and transport, initiation and extension of translation process, regulation of translation rate and direct influence the stability of mRNA itself (Wan et al., 2011; Dethoff et al., 2012). The base change of SNP may change the secondary structure of mRNA, so we used RNAfold to predict the secondary structure of mRNA, and MFE was used as an indicator to measure the stability of the secondary structure in this study. Five sites, 3:g.15345216T, 3:g.15349768G, 3:g.15350898C, 3:g.15353235C, and 3:g.15353254C, with higher MFEs compared that to the reference sites, caused the instability of *PKLR* mRNA secondary structure to inhibit its expression, additionally, our study found that the five loci were significantly associated with milk fat yield, and the phenotypic value of fat yield of homozygous individuals at the mutation site was significantly reduced. On the contrary, three sites, 3:g.15353292G, 3:g.15353330G, and 3:g.15353342T, had lower MFEs and more stable mRNA structure, also had significant genetic effects on fat yield, and the phenotypes of fat yield of homozygous cows at these sites were significant increased. It suggested that these eight SNP sites might affect milk fat yield of dairy cows by influencing the instability of mRNA secondary structure of *PKLR*. Further, we speculated that the changes of mRNA secondary structures caused by SNPs may affect the stability of its higher-order structure and gene expression, leading to an influence on milk production phenotypes of dairy cows.

## Conclusion

In summary, a total of 21 SNPs were identified in *PKLR* gene, and their significant genetic associations with milk production traits of dairy cows have been confirmed. Eleven SNPs might be the potential causal mutations for the milk production traits in dairy cattle that needs more in-depth validation, of which, 3:g.15342877C>T, 3:g.15344349A>C, 3:g.15345216C>T, and 3:g.15345227T>C might change the TFBSs to regulate expression of the *PKLR* gene, and eight SNPs, 3:g.15345216C>T, 3:g.15349768A>G, 3:g.15350898T>C, 3:g.15353235T>C, 3:g.15353254T>C, 3:g.15353292C>G, 3:g.15353330A>G, and 3:g.15353342C>T, could change the secondary structure of mRNA and the phenotypic value of fat yield. The valuable SNPs could be used as candidate genetic markers for dairy cattle molecular breeding for the development of GS chip.

## Data Availability

The original contributions presented in the study are included in the article/Supplementary Material, further inquiries can be directed to the corresponding author.
